# Comprehensive RNA-Seq Profiling to Evaluate the Sheep Mammary Gland Transcriptome in Response to Experimental *Mycoplasma agalactiae* Infection

**DOI:** 10.1371/journal.pone.0170015

**Published:** 2017-01-12

**Authors:** Rohini Chopra-Dewasthaly, Melanie Korb, René Brunthaler, Reinhard Ertl

**Affiliations:** 1 Division of Clinical Microbiology and Infection Biology, Institute of Microbiology, Department of Pathobiology, University of Veterinary Medicine, Veterinaerplatz 1,Vienna, Austria; 2 VetCore Facility for Research, University of Veterinary Medicine, Veterinaerplatz 1,Vienna, Austria; 3 Institute of Pathology and Forensic Veterinary Medicine, Department of Pathobiology, University of Veterinary Medicine, Veterinaerplatz 1, Vienna, Austria; The University of Melbourne, AUSTRALIA

## Abstract

*Mycoplasma agalactiae* is a worldwide serious pathogen of small ruminants that usually spreads through the mammary route causing acute to subacute mastitis progressing to chronic persistent disease that is hard to eradicate. Knowledge of mechanisms of its pathogenesis and persistence in the mammary gland are still insufficient, especially the host-pathogen interplay that enables it to reside in a chronic subclinical state. This study reports transcriptome profiling of mammary tissue from udders of sheep experimentally infected with *M*. *agalactiae* type strain PG2 in comparison with uninfected control animals using Illumina RNA-sequencing (RNA-Seq). Several differentially expressed genes (DEGs) were observed in the infected udders and RT-qPCR analyses of selected DEGs showed their expression profiles to be in agreement with results from RNA-Seq. Gene Ontology (GO) analysis revealed majority of the DEGs to be associated with mycoplasma defense responses that are directly or indirectly involved in host innate and adaptive immune responses. Similar RNA-Seq analyses were also performed with spleen cells of the same sheep to know the specific systemic transcriptome responses. Spleen cells exhibited a comparatively lower number of DEGs suggesting a less prominent host response in this organ. To our knowledge this is the first study that describes host transcriptomics of *M*. *agalactiae* infection and the related immune-inflammatory responses. The data provides useful information to further dissect the molecular genetic mechanisms underlying mycoplasma mastitis, which is a prerequisite for designing effective intervention strategies.

## Introduction

Mycoplasmas are one of the smallest and simplest microbes that cause difficult-to-eradicate chronic infections by complex unknown pathogenicity factors [1, 2]. Mycoplasma mastitis is one such worldwide problem. Amongst different causative species *Mycoplasma bovis* is the most important species in cattle, whereas its very close phylogenetic relative *Mycoplasma agalactiae*, inflicts similar severe mastitis in small ruminants by complex immuno-inflammatory pathophysiological processes that are yet to be fully comprehended [3–5]. *M*. *agalactiae* is the main etiological agent of contagious agalactia (CA) syndrome in sheep and goats and causes major economic losses, especially due to its persistence and shedding in milk for many years even after antibiotic treatment of the initial infection [6–9]. Its socio-clinical significance can be stressed by the fact that unlike the economically important *M*. *bovis* infections, CA is enlisted as a notifiable disease by the World Organization for Animal Health [10].

Despite its importance, the complex host-pathogen interactions of *M*. *agalactiae* enabling survival in the mammary gland are not completely understood. We had recently identified potential pathogenicity factors by negative selection of transposon mutants that were unable to survive in the experimentally infected udders [11, 12]. Although the exact role of most of the identified factors remains to be elucidated, pyruvate dehydrogenase was shown to be involved in cell invasiveness [13], a strategy that *M*. *agalactiae* likely employs to invade mammary cells to avoid antibiotics and host defenses, and to spread systemically to new host niches [14, 15]. Another study has described neutrophil extracellular trap (NET) formation *in vivo* in the mammary gland and milk of sheep naturally infected with *M*. *agalactiae* [16]. Although mycoplasma liposoluble proteins are demonstrated to induce NET release, the exact role of the recently identified phase variable β-(1→6)-Glucan is yet to be elucidated in its disease progression [17].

With respect to the involved host factors, except for a couple of immune response reports, that too in goats, where under natural conditions four other mycoplasma species are known to cause clinically indistinguishable syndrome [4, 18], the dynamics of *M*. *agalactiae* induced host responses in the mastitic sheep mammary gland are almost completely unknown. One study has described proteomic changes occurring in milk fat globules (MFG) during sheep infectious mastitis [19]. Although MFGs can sometimes provide important information, yet this indirect approach is often incomplete in defining the mastitic mammary tissue. For instance, only some of the host response proteins identified in bovine mastitis have been shown to be associated with MFGs [20, 21]. Here, we have tried to address this issue by studying the transcriptional responses within ovine udders subjected to intramammary infection with *M*. *agalactiae* type strain PG2. In addition to the udder transcriptome, we also analyzed gene expression changes in the spleen to get an idea about systemic responses away from the site of infection. The up and down regulation of host factors, especially the innate and adaptive immune responses, would be instrumental in determining the level of immunmodulation, and thus the susceptibility of the mammary gland to *M*. *agalactiae* infection to progress to a subacute or chronic disease while being excreted in the milk.

## Material and Methods

### Inoculum

*M*. *agalactiae* pathogenic type strain PG2 [22, 23] was grown at 37°C in SP4 medium supplemented with penicillin, pyruvate, and phenol red as indicator as described before [24]. The inoculum for sheep experiment was prepared as described earlier [25] with slight modifications. Cultures were centrifuged at 10000 g for 15 min at 4°C, and pellets washed with 1X Phosphate Buffered Saline (PBS) (Gibco® by Life Technologies) before final resuspension and storage at -80°C as 500 μl aliquots. The titre of viable mycoplasmas was determined prior to inoculation day by plating serial ten-fold dilutions of two different aliquots as described earlier [25]. Fresh aliquots were accordingly pooled to get an inoculum size of 10^9^ cfu per sheep in 5 ml of PBS. A common pooled inoculum with some extra volume was prepared for all sheep to be infected and the residual volumes were used for plating 10-fold serial dilutions to verify the actual viable counts injected per animal.

### Animals and intramammary infection model

Six healthy lactating sheep of the local mountain breed were used for this experiment. All of them were free of major sheep pathogens, and were also confirmed to be seronegative for *M*. *agalactiae* by a commercial EIA kit (IDEXX *M*. *agalactiae* Ab test kit). This also negated any previous contact with *M*. *agalactiae*. Three sheep received 10^9^ cfu of mycoplasmas in 5 ml of PBS in the right teat canal. The three control sheep were instead inoculated with 5 ml of PBS. Standard clinical examinations were performed as recommended by Baumgartner et al. [26]. Sheep were euthanized after about 2 weeks of infection to obtain udder and spleen samples. The biopsies were minced and placed in RNALater Stabilization Reagent (Qiagen) and snap-frozen in liquid nitrogen before storing at -80°C.

### Ethic statement

The sheep experiment was approved by the Ethics and Animal Welfare Commission of the University of Veterinary Medicine Vienna and the Austrian Federal Ministry for Science, Research and Economics (approval number: BMWFW-68.205/0106-WF/II/3b/2014). The animals were housed in the stables at the University of Veterinary Medicine Vienna and all procedures were carried out in accordance with the institutional ethics committee. The sheep were anaesthetized by Thiopental before euthanizing them via the intravenous (*Vena jugularis*) injection of T61 as recommended for sheep in the drug information (Austria Codex).

### Histology and immunohistochemical analysis

Tissue samples were fixed in 10% buffered formalin and alcohol dehydrated before embedding in paraffin wax. Subsequently, paraffin sections (5 μm) were cut and stained with hematoxylin-eosin and examined by light microscopy. Immunohistochemical analysis was performed as described earlier [14] using *M*. *agalactiae*-specific rabbit polyclonal antiserum.

### RNA extraction

Tissues were placed in 600 μl RLT buffer (Qiagen) supplemented with 6 μl β-mercapthoethanol and mechanically homogenized on a MagNA Lyser instrument (Roche Diagnostics, Rotkreuz, Switzerland) using 1.4 mm ceramic beads at 6500 rpm for 25 sec. Total RNA was extracted using the RNeasy Mini Kit (Qiagen) following the recommended protocol for animal tissues. Two replicate RNA extractions were performed for each of the three animals, resulting in a total of 12 udder and 12 spleen samples (6 non-infected, 6 *M*. *agalactiae*-infected). The RNA Clean & Concentrator-5 Kit (Zymo Research, Irvine, USA) was used for DNase treatment, RNA purification and the removal of digested DNA. RNA quality was assessed by capillary electrophoresis on the Agilent 2100 Bioanalyzer using the RNA 6000 Nano Kit (Agilent Technologies, Santa Clara, USA). The obtained RNA integrity numbers (RINs) are enlisted in the S1 Table. RNA concentrations were measured by UV spectrophotometry on the NanoDrop 2000c (Thermo Scientific, Waltham, USA).

### RNA-sequencing and statistics for differentially expressed genes

1 μg of total RNA per sample was used as starting material for the preparation of mRNA libraries utilizing the TruSeq RNA Sample Prep Kit v2 (Illumina, San Diego, USA) according to the manufacturers’ instructions. Sequencing was performed by the Next Generation Sequencing (NGS) unit of the Vienna Biocenter Core Facilities GmbH (VBCF, Vienna, Austria). A total of 24 mRNA libraries were sequenced on two lanes of an Illumina HiSeq 2500 NGS system to generate 50 bp single-read sequences. Data analysis was performed essentially as described earlier [27]. Briefly, the Trimmomatic trimming tool was used for quality control and filtering of the sequencing data [28]. All bases below an Illumina quality score of 25 and contaminating adapter sequences were removed. Reads shorter than 25 bp were discarded. The filtered reads were then mapped against sheep cDNA reference sequences (release 78) from Ensembl database (ftp://ftp.ensembl.org/pub/release-78/fasta/ovis_aries/cdna/) with the QSeq v5 RNA-Seq analysis software (DNASTAR, Madison, USA) using the recommended mapping parameters for Illumina short reads (matching of a minimum of 20 bases and at least 80% of the bases within each read). Out of the filtered reads, 63.01–72.7% and 74.32–76.93% could be mapped to the reference for mammary gland and spleen samples, respectively. Numbers of mapping reads per transcript were counted and differentially expressed genes (DEGs) were identified by comparing the mean values of the *M*. *agalactiae*-infected samples with the respective non-infected control tissues. Genes with fold changes > 2 and p-values ≤ 0.01 were considered differentially expressed. The p-values were adjusted for multiple testing (false discovery rate, FDR) with the Benjamini-Hochberg correction method using a cut-off value of ≤ 0.05 [29, 30].

Transcripts that were specified as ‘novel genes’ on the current version of the sheep reference genome were used for a blast search in NCBI RefSeq database (http://www.ncbi.nlm.nih.gov/refseq/) to identify the gene names. The sequences for which no annotation was available for sheep (*Ovis aries)*, gene names of orthologous species with the highest sequence identity were used for down-stream analysis. The sequencing reads have been submitted to the European Nucleotide Archive (www.ebi.ac.uk/arrayexpress/) under the accession number E-MTAB-5216.

### Functional categorization

The generated list of *M*. *agalactiae*-induced DEGs was analyzed for significantly enriched biological processes using the ClueGO v2.2.4 plugin [31] of the biomolecular network software platform Cytoscape v3.1.1 [32]. Due to the lack of Gene Ontology (GO) data for sheep (*Ovis aries*), the biological processes were analyzed based on human GO data. All enriched GO terms for biological processes that possessed a p-value ≤ 0.01 are displayed.

### Reverse transcription quantitative PCR

RT-qPCR was performed to verify the RNA-Seq results for 5 DEGs found in the mammary gland and 2 DEGs in the spleen. Corresponding RT-qPCR primers for five up- (CXCL13, IDO1, CCL19, Natural killer cells antigen CD94-like [NK CD94] and NPTX1) and two down-regulated target genes (LALBA and OBP2B) were designed using the PrimerExpress 2.0 software (Life Technologies, Foster City, USA). All assays were validated by the generation of standard curves for calculating PCR efficiencies (E) using the formula: E = −1 + 10^(−1/slope)^ [33]. Detailed assay data are listed in Table 1. OAZ1 and RPL27, encoding Ornithine decarboxylase antizyme 1 and Ribosomal protein L27, respectively, were used as reference genes for normalization. The expression stability of both reference genes was assessed using the RefFinder tool (http://fulxie.0fees.us/?type=reference) [34]. For RT-qPCR, 500 ng total RNA was converted into cDNA using the High Capacity Reverse Transcription Kit (Life Technologies) according to the recommended protocol. Controls without RT-enzyme were included for each sample to monitor the amplification of residual DNA. RT-qPCR was performed with the fluorescent DNA dye EvaGreen (Biotium, Hayward, USA) using the reaction conditions described earlier [27]. All samples were analyzed in duplicates on a Viia7 Real-Time PCR System (Life Technologies). Target gene expression levels were normalized to the geometric mean of both reference genes and relative expression changes were calculated using the comparative 2^-ΔΔCT method [35]. Statistical analysis (Mann-Whitney *U* test) of the RT-qPCR data was performed with GraphPad Prism 5 (GraphPad Software, La Jolla, USA). A p-value < 0.05 was considered significant.

**Table 1:** Details of RT-qPCR assays used for validation of RNA-Seq results.

Accession number*	Gene symbol	Gene name	Oligo	Sequence (5’– 3’)	Amplicon size (bp)	PCR efficiency (%)
XM_004004120.3 XM_012158653.2	*CCL19*	Chemokine (C-C motif) ligand 19	Forward	GGGTGGGCCGCATCA	97	91.20
			Reverse	CGGAACACAGGGCTCTCTCT		
XM_012122842.1	*CXCL13*	Chemokine (C-X-C motif) ligand 13	Forward	GCGGGAACTCCACCTTGA	97	101.35
			Reverse	CCAGAACACCGTGGACAGG		
NM_001141953.1	*IDO1*	Indoleamine 2,3-dioxygenase 1	Forward	GATACATCACCATGGCGTATGTGT	135	91.40
			Reverse	AATCCGCATAAAGCAGAATAGGA		
NM_001009797.1	*LALBA*	Lactalbumin, alpha	Forward	ACCGCATTTCATACCAGTGGTTA	158	90.63
			Reverse	AGGAACTTGTCACAGGAGATGTTACA		
XM_012175399	*N/A*	Natural killer cells antigen CD94-like	Forward	GACCCTCCACAGATCTCAGTGAA	121	92.34
			Reverse	TCCCTACTGTCTTTCCATGTTTTTAATT		
XM_004013084.3	*NPTX1*	Neuronal pentraxin I	Forward	GGTCCTCATTGAGTGGGGC	91	94.54
			Reverse	CCACTTGCCGTCATTGATTACA		
NM_001289795.2	*OAZ1*	Ornithine decarboxylase antizyme 1	Forward	TTGCTTCCACAAGAACCGC	75	93.98
			Reverse	CTCACAATCTCAAAGCCCAAA		
XM_012175883.2	*OBP2B*	Odorant binding protein 2B	Forward	CCCGCTGCCATGAGAAGA	71	106.31
			Reverse	GCCGCCATTGGAGCTGTA		
XM_015098799.1 XM_012152407.2 XM_012152408.1	*RPL27*	Ribosomal protein L27	Forward	GCCCGACGAGAGGCAAA	76	90.41
			Reverse	GCAGCTTCTGGAAGAACCATT		

*National Center for Biotechnology Information (NCBI), Entrez Gene [http://www.ncbi.nlm.nih.gov/sites/entrez?db=gene]

## Results

### Establishment of *M*. *agalactiae* mastitis

**Fig 1:**
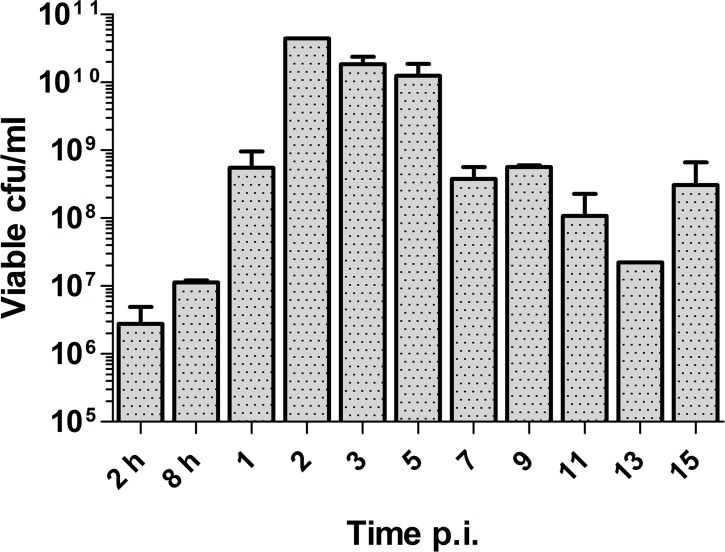
Continuous shedding of *M*. *agalactiae* in milk from mastitic right udders. Time-course of *M*. *agalactiae* shedding in milk from inoculated right udder halves. Mean log_10_ cfu values of viable mycoplasmas per milliliter of milk are depicted and bars represent standard deviation of the mean.

Milk samples from all sheep were negative for mycoplasmas and other major sheep pathogens when tested before infection. After intramammary inoculation of *M*. *agalactiae* in the right teat canal, the pathogen was able to successfully multiply and colonize the right udder halves, and in one sheep also in the non-inoculated left udder half. *M*. *agalactiae* was continuously shed in the milk from the infected udders soon after 2 h of infection (4.00 x 10^6^ cfu/ml) until the end of the experiment, that is, until Day 15 p.i. (3.08 x 10^8^ cfu/ml), although peak cfu values were reached on Day 2 p.i (4.44 x 10^10^ cfu/ml; Fig 1). Except for one sheep that showed positive isolation of *M*. *agalactiae* starting from Day 7 p.i., the milk from left udder halves was negative for mycoplasmas at all tested time points, as was also the case with non-infected control sheep for both udder halves. Besides being positive for California Mastitis Test, both the quantity and quality of milk from right udder halves was severely affected, ranging from no milk to grey watery secretions, sometimes with flocks and/or blood.

**Fig 2:**
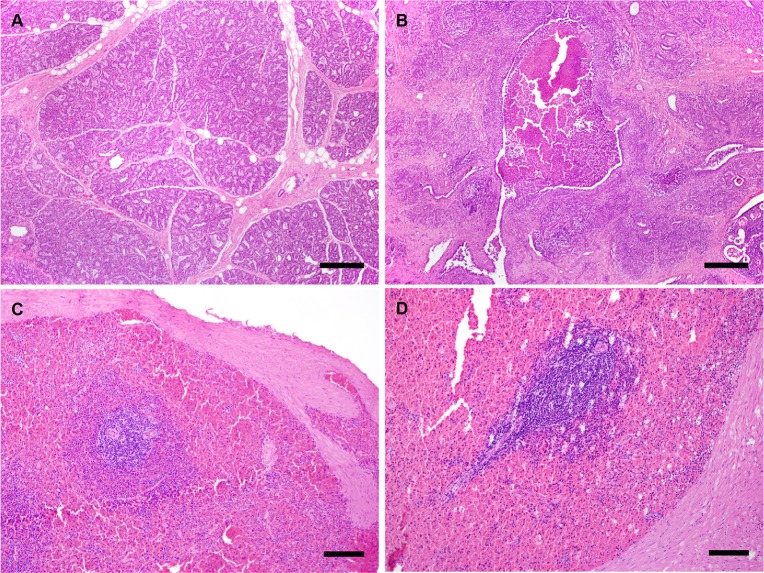
Histological sections of udder and spleen. Histological changes in the udder of a sheep experimentally infected with *M*. *agalactie* (B) compared to uninfected control animal (A). Spleen sections from both infected (D) and uninfected control (C) demonstrated no major differences. Haematoxylin-eosin stained preparations. *Scale bar* 400 μm (A and B) and 150 μm (C and D).

**Fig 3:**
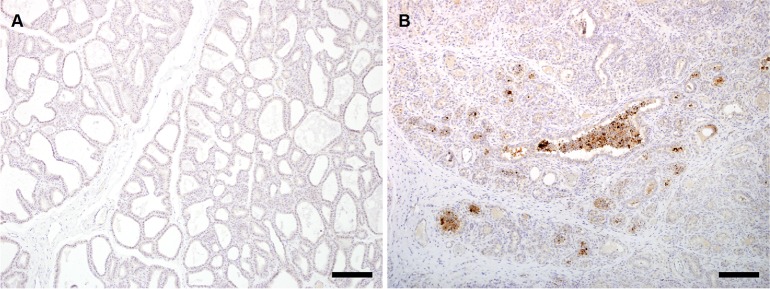
Immunohistochemical analysis of sheep udders. Detection of *M*. *agalactiae* as positive signals (brown staining) in the immunohistochemically stained sections of udders of sheep experimentally infected via the intramammary route (B). Corresponding section from uninfected control animal shows no positive staining (A). Bar = 150 μm.

The right udder halves of all infected sheep were clearly thicker and more firm compared to the uninfected udders and histologically demonstrated a typical pathomorphological picture of mycoplasma mastitis that was absent in the uninfected control animals (Fig 2). For instance, cross sections revealed multiple micro abscesses and a significant proliferation of the inter- and intra- lobular interstitial connective tissue with focal accumulations of lymphocytes, histiocytes, plasma and mast cells (Fig 2B) indicating a middle grade non-purulent interstitial mastitis with atrophy of the glandular tissue and oligofocal purulent galactaphoritis. The udders from non-infected control animals appeared normal (Fig 2A). Furthermore, *M*. *agalactiae* antigens were detected in the endothelium, epithelium, as well as in histiocytes when infected udder samples were subjected to immunohistohemical analysis using *M*. *agalactiae-*specific antiserum. Fig 3 illustrates representative negative (Fig 3A) and positive (Fig 3B) immunohistochemically stained sections from control (uninfected) and infected udder samples, respectively.

Spleen samples did not reveal any significant differences between the infected and uninfected control animals as demonstrated in Fig 2C and 2D.

### Differential gene expression analyses of *M*. *agalactiae*-infected mammary gland compared to uninfected healthy control tissue

To better understand the host responses occurring during mycoplasma mastitis, six different mammary gland tissue samples, consisting of biological replicates from three different infected sheep were subjected to mRNA deep sequencing and compared with six similar non-infected sheep samples as described under Materials and Methods. Using stringent quality control and filtering parameters for the sequenced data, and considering p-value significance of only less than or equal to 0.01, we identified 39 DEGs in the mastitic udder tissue, out of which 36 were up-regulated (Table 2). Most of the identified DEGs encode proteins that are directly or indirectly involved in host defense against the pathogen either indirectly by changing the physiological status of the udder or milk or by inducing robust immune responses (Table 2) leading to inflammation. Most conspicuous is the more than hundred fold upregulation of two genes, namely WFDC18 (133-fold) and CXCL13 (121-fold), encoding an extracellular proteinase inhibitor and a B-cell attracting chemokine, respectively. Although a pathogenetic role has been reported for CXCL13, both in mycoplasma and non-mycoplasma diseases [36–38], the upregulation of WFDC18 is interesting and needs to be thoroughly studied. This is because proteases often constitute one of the primary targets in drug discovery and extracellular proteinases are known to be differentially expressed in pathophysiological processes like cancer and inflammation [39].

**Table 2:** List of DEGs in sheep mammary gland after *M*. *agalactiae* infection. Fold changes (*M*. *agalactiae* vs. uninfected) display the mean value of 6 replicate samples obtained from 3 sheep per group. Genes were ranked according to the expression changes (up- or down-regulation) detected by RNA-Seq.

**Down-regulated genes**				
**Gene symbol**	**Description**	**Fold change**	**P-value**	FDR*
OBP2B	Odorant binding protein 2B	-144.78	9.22E-07	3.67E-03
PRG3	Proteoglycan 3-like (LOC101105274)	-57.28	8.20E-05	4.80E-02
LALBA	Lactalbumin, alpha	-38.08	5.62E-05	4.07E-02
**Up-regulated genes**				
**Gene symbol**	**Description**	**Fold change**	**P-value**	FDR*
WFDC18	Extracellular proteinase inhibitor	133.22	4.20E-09	8.37E-05
CXCL13	Chemokine (C-X-C motif) ligand 13	120.99	4.56E-08	4.54E-04
LCN1	Lipocalin 1	87.70	6.72E-07	3.67E-03
IDO1	Indoleamine 2,3-dioxygenase 1	85.65	1.05E-05	1.35E-02
RETN	Resistin	72.08	6.13E-06	1.22E-02
TRDC	T cell receptor delta constant	70.93	2.86E-05	2.72E-02
APOBEC3Z1	Apolipoprotein B mRNA editing enzyme, catalytic polypeptide-like	62.73	5.72E-05	4.07E-02
OLA-I	BOLA class I histocompatibility antigen, α chain BL3-7-like	62.60	9.14E-05	4.84E-02
LYZ	Lysozyme	61.12	4.32E-05	3.74E-02
MYL4	Myosin light chain 4	57.11	6.64E-05	4.41E-02
CD79B	CD79b molecule	49.06	7.99E-06	1.33E-02
C7H15orf48	Chromosome 7 open reading frame, human C15 orf48	42.31	4.07E-06	1.09E-02
IGHG1	Immunoglobulin heavy constant gamma 1	35.06	7.46E-07	3.67E-03
DQA1	SLA class II histocompatibility antigen, DQ haplotype D α chain	34.47	9.43E-05	4.84E-02
IGLV2	Immunoglobulin lambda-2c light chain variable region	33.43	6.51E-05	4.41E-02
ZG16B	Zymogen granule protein 16B	30.33	4.55E-06	1.09E-02
LOC102332574	Uncharacterized protein	29.26	3.27E-06	1.09E-02
ORM1	Orosomucoid 1	27.41	1.44E-05	1.62E-02
LOC100101238	Regakine 1-like protein	27.04	8.73E-06	1.34E-02
IGHG2	Immunoglobulin gamma 2 constant region heavy chain	25.95	4.93E-06	1.09E-02
PTPRCAP	Protein tyrosine phosphatase, receptor type, C-associated	25.54	1.88E-05	1.97E-02
LTB	Lymphotoxin beta	25.20	1.09E-05	1.35E-02
TMEM176A	Transmembrane protein 176A	25.04	5.70E-05	4.07E-02
IGG2C	Ig germline heavy chain gamma-2-chain gene C-region, 3' end	24.62	7.42E-05	4.48E-02
LEPREL4	Leprecan-like 4	24.40	6.92E-05	4.45E-02
CCL19	Chemokine (C-C motif) ligand 19	23.47	9.45E-06	1.34E-02
MZB1	Marginal zone B and B1 cell-specific protein	23.28	7.73E-06	1.33E-02
CD19	CD19 molecule	23.19	9.11E-05	4.84E-02
S100A12	S100 calcium binding protein A12	22.51	1.46E-05	1.62E-02
IGLV3	Immunoglobulin V lambda chain (V lambda 3) gene	19.45	9.12E-05	4.84E-02
IGK	Immunoglobulin kappa chain	18.85	4.81E-05	3.84E-02
ADA	Adenosine deaminase	18.45	7.23E-05	4.48E-02
IGLC1	Immunoglobulin lambda constant 1	17.25	4.27E-05	3.74E-02
TR-BETATRB	T-cell receptor beta chain T17T-22-like (LOC101107504)	16.92	9.49E-05	4.84E-02
S100A8	S100 calcium binding protein A8	15.67	4.82E-05	3.84E-02
CD86	CD86 molecule	14.82	9.71E-05	4.84E-02

*FDR: False discovery rate

Also, the significant down-regulation of fat and milk-associated proteins, namely OBP2B (145-fold) and LALBA (38-fold) is quite remarkable. The complete list of all the DEGs showing 15- to 145-fold difference in their expression is enlisted in Table 2.

**Fig 4:**
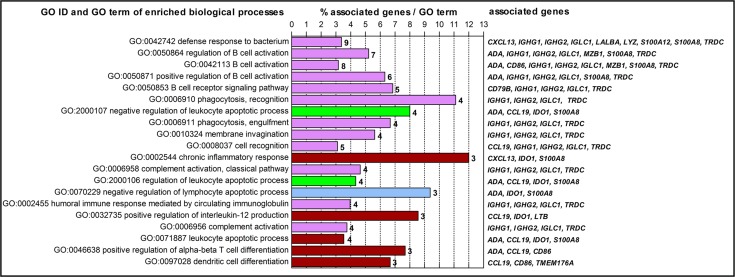
Top 20 enriched Gene Ontology (GO) biological processes in *M*. *agalactiae*-infected mammary gland. The list of DEGs detected by RNA-Seq was investigated for significantly (p ≤ 0.01) enriched biological processes using the ClueGO biomolecular network tool. All enriched biological processes were ranked from top to bottom according to the p-values for each GO term. Bars represent the percentage of associated genes found regulated in *M*. *agalactiae*-infected mammary glands relative to the total number of genes assigned to each GO term. Colors reflect the degree of connectivity between terms based on the similarity of the associated genes. Absolute numbers of associated genes are depicted at the end of the bars.

When analyzed for the significantly enriched (p-value ≤ 0.01) biological processes using the ClueGO v2.2.4 plug-in [31] and the biomolecular network software Cytoscape v3.1.1 [32], most of the DEGs were directly or indirectly associated with host defense response against the mycoplasma pathogen (Fig 4 and S2 Table). The highest number of identified genes were classified directly under defense response (GO:0042742), whereas others were involved with immune responses, such as B cell activation and its regulation, chronic inflammatory response, phagocytosis, complement activation, positive regulation of interleukin 12 production and T alpha-beta and dendritic cell differentiation (Fig 4).

### Less prominent differential gene expression in spleen cells of *M*. *agalactiae*-infected sheep

In order to assess the possible systemic changes occurring in an internal site distant to the local infection area, 6 different spleen tissues from *M*. *agalactiae*-infected sheep were compared with similar tissues obtained from uninfected sheep. Differential gene expression was overall down-sized in the spleen cells, both in terms of number of identified DEGs, as well the fold difference in expression profile compared to the spleens obtained from the uninfected controls. The genes are enlisted in Table 3. Regarding the biological significance of the DEGs identified in spleen, the GO analysis did not yield any conclusive results as no processes or pathways were found to be significantly enriched.

**Table 3:** List of DEGs in sheep spleen after *M*. *agalactiae* infection. Fold changes (*M*. *agalactiae* vs. uninfected) display the mean value of 6 replicate samples obtained from 3 animals per group. Genes were ranked according to the expression changes (up- or down-regulation) detected by RNA-Seq.

**Down-regulated genes**				
**Gene symbol**	**Description**	**Fold change**	**P-value**	FDR*
HNRNPDL	Heterogeneous nuclear ribonucleoprotein D-like	-3.65	1.71E-04	3.33E-02
NQO1	NAD(P)H dehydrogenase, quinone 1	-3.35	1.03E-04	3.13E-02
LOC105616883	L-xylulose reductase	-3.27	7.62E-04	4.31E-02
DDC	Dopa decarboxylase	-3.05	3.28E-04	3.52E-02
SLC48A1	Solute carrier family 48 (heme transporter), member 1	-2.22	7.22E-04	4.29E-02
SDC1	Syndecan 1	-2.08	7.99E-04	4.40E-02
LOC101120019	60S ribosomal protein L10a-like	-2.01	6.64E-04	4.15E-02
**Up-regulated genes**				
**Gene symbol**	**Description**	**Fold change**	**P-value**	FDR*
LOC101102227	Natural killer cells antigen CD94-like	4.37	7.86E-04	4.37E-02
OLA-I	BOLA class I histocompatibility antigen, alpha chain BL3-7-like	3.64	5.70E-04	4.01E-02
NPTX1	Neuronal pentraxin I	3.60	3.67E-05	2.48E-02
LOC101121646	T-lymphocyte surface antigen Ly-9	3.55	8.89E-04	4.65E-02
STYK1	Serine/threonine/tyrosine kinase 1	3.52	7.80E-04	4.36E-02
NLRP10	NLR family, pyrin domain containing 10	2.75	7.22E-04	4.29E-02
LOC101106498	NKG2D ligand 1-like	2.70	8.17E-04	4.44E-02
LOC101118241	Testisin-like	2.61	5.50E-04	3.97E-02
SLC26A7	Solute carrier family 26 (anion exchanger), member 7	2.51	9.44E-05	3.11E-02
FREM1	FRAS1 related extracellular matrix 1	2.50	4.01E-04	3.64E-02
CPXM1	Carboxypeptidase X (M14 family), member 1	2.42	4.25E-04	3.68E-02
KIR3DP1	Putative killer cell immunoglobulin-like receptor like protein	2.31	1.71E-04	3.33E-02
LOC105605896	Musimon fibrous sheath-interacting protein 2-like	2.23	7.26E-04	4.29E-02
TFRC	Transferrin receptor	2.15	1.82E-04	3.33E-02
EEF1A1	Elongation factor 1-alpha 1	2.05	4.64E-04	3.78E-02

*FDR: False discovery rate

### Validation of RNA-Seq results by quantitative real time PCR

**Fig 5:**
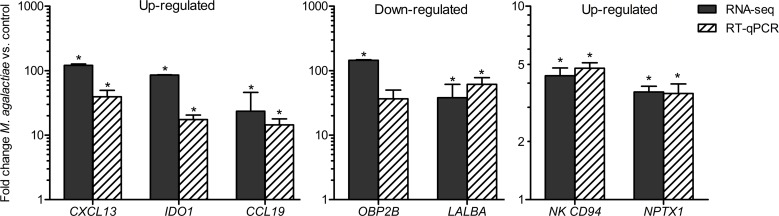
Validation of RNA-Seq results by RT-qPCR. Fold changes (*M*. *agalactiae* vs. non-infected) obtained by RT-qPCR were compared with the sequencing results for seven DEGs. Bars represent the mean values ± SEM of 6 replicates per group. Stars indicate significant expression changes.

RNA sequencing results were validated by performing RT-qPCR for seven DEGs, out of which five were differentially expressed in the mammary gland (CXCL13, IDO1, CCL19, LALBA, OBP2B) and two in spleen (NK CD94, NPTX1). As shown in Fig 5, the relative gene expression levels of selected genes measured by RT-qPCR were in good agreement with the sequencing data, that is, CXCL13, IDO1, CCL19, NK CD94 and NPTX1 showed increased expression in the respective tissues, whereas LALBA and OBP2B showed a decrease in expression in the mammary gland. Also, except for the latter gene, all others showed a significant expression change (p-value <0.05) via RT-qPCR analysis. RT-qPCR data (mean Cq-values of technical replicates) are enclosed in S1 Table.

## Discussion

The primary objective of this study was to characterize the host transcriptional response to mycoplasma infections of ruminants. More specifically, the ovine response was analyzed by RNA deep sequencing of mammary gland tissue of three lactating sheep after an experimental intramammary infection with *M*. *agalactiae*. Gene expression profiling has been intensively used for several mycoplasma species to study host-pathogen interactions. In this context, the transcriptomes of either the host or the pathogen were investigated to gain further insights into mycoplasma-induced pathogenesis [40–44]. To the best of our knowledge this is a singular study in many aspects. Firstly, there is hardly any literature on the mammary gland transcriptome analysis of mycoplasma mastitis infections, although one report has indirectly evaluated sheep responses by using MFG proteomics [19]. Secondly, unlike for bovine infections, transcriptome analysis of sheep mammary gland, per se, has never been thoroughly described in response to even any bacterial pathogen causing mastitis except for a couple of reports that used milk somatic cell and bone marrow-derived dendritic cell transcriptomics, respectively [45–47]. Thirdly, the study utilizes the more powerful deep sequencing strategy to identify DEGs instead of the more commonly used microarray datasets. Lastly, here the spleen transcriptome was also evaluated together with the mammary response to intramammary mycoplasma challenge in order to understand the systemic immune responses in a peripheral tissue. Most of the other studies evaluated the liver or adipose cell transcriptomes to understand the systemic responses to mammary gland infections [47] and spleen responses are hardly studied during mastitis.

The study identified a relatively lower number of DEGs compared to other reports utilizing different approaches to describe similar responses in different hosts and different intramammary pathogens [21, 47–49]. Apart from other differences, this could be partially attributed to the highly stringent selection criteria used for selecting DEGs and partially to the mild self-contained infection elicited during the experimental conditions and genetic background of the sheep, which have been known to be important parameters for evoking responses [45]. Also, animal tissues are composed of a mixture of different cell types with varying infection rates and host cell responses, resulting in a less uniform gene expression pattern in the complete tissue and lower numbers of DEGs compared to respective *in vitro* studies. The DEGs detected in the present study, however, show a distinct response in mixed samples of the natural host and can be considered as essential cellular factors involved in *M*. *agalactiae* pathogenesis *in vivo*.

When analyzed in the context of the biological pathways and gene ontologies, most of the identified DEGs were significantly (p-value ≤ 0.01) associated with robust host defense responses against the mammary pathogen as shown in Fig 4. Several biological processes involving B cells as well as processes associated with phagocytosis, such as cell recognition, engulfment and membrane invagination were among the significantly enriched GO terms (Fig 4). Taken together, these results indicate the state of a chronic infection with increased activity of immune cells, in particular, B cells and macrophages. The host defense is accomplished either directly via upregulating the innate response genes encoding proteins like lipocalin (88-fold increase) and lysozyme (61-fold increase), or indirectly through the induction of chemokines like CXCL13 (121-fold increase) and CCL19 (24-fold increase), or by activation of B cells and the complement system.

Another important feature of this host response was the induction of genes involved in immunosuppression, such as ORM1 encoding Orosomucoid 1 (27-fold increase) or immunomodulation via genes involved in the positive regulation of IL-12, a pro-inflammatory cytokine, namely by IDO1 (86-fold increase), LTB and CCL19 overexpression. These results corroborate with the fact that many mycoplasmas are known to first stimulate the innate immune system, including intricate signaling systems that include cytokines and chemotactic molecules, to attract and activate leucocytes, and subsequently activate cells of the adaptive immune system [37]. *M*.*agalactiae* has also been previously reported to induce a strong innate immune response involving neutrophils and macrophages in the first few days of infection in goats, subsequently followed by a specific antibody response by Day 7 p.i. that coincided with reduced viable mycoplasma counts in milk, but showed this humoral response to be incapable of controlling mycoplasma invasion [4].

Another significant feature of the mammary host response was the significant enrichment of the DEGs involved in chronic inflammatory response (CXCL13, IDO1 and S100A8) (Fig 4) that led to the mastitis phenotype. Other identified DEGs, such as S100A12 and APOBEC3Z1 are also known to modulate the inflammatory response and have been previously identified in similar bovine mammary gland infections [21, 50]. Furthermore, members of the S100 protein family, such as S100A8 and S100A12, are also involved in the host cell response to human and porcine pathogens, such as *M*. *genitalum*, *M*. *hominis* and *M*. *hyopneumoniae* [51–53]. These small calcium-binding proteins interact with the host immune system and the pathogen, and are reported to play an important role during infection and inflammation [54]. This is in agreement with the pathogenicity of mycoplasmas, which is often attributed to the immunopathological host responses evoked by them and their atypical characteristics have often been associated with these dysregulated immune responses [37]. A previous experimental *M*. *agalactiae* infection study in goats has correlated the immune response with the clinical-pathological course of persistent mastitis [4] and immunoinflammatory response has been chronologically characterized via immunohistochemistry [18].

Although most of the DEGs showing increased expression are associated with immuno-inflammatory and other host defense responses, two out of the three significantly down-regulated genes, namely OBP2B (145-fold), and LALBA (38-fold) encoding odorant binding protein 2B and lactalbumin alpha, respectively, are proteins implicated in lipid metabolism/transport or major milk proteins affecting milk quality and quantity as also observed in other studies where down-regulated genes were mostly involved with ontologies ‘lactation’ and ‘lipid metabolism’ [19, 47, 49, 55]. The third downregulated gene PRG3 (57-fold) encodes proteoglycan 3. This is interesting as the latter is known for its cytotoxic and cytostimulatory activities, whereby it has been shown to stimulate neutrophil superoxide and IL-18, and histamine and leukotrienes from basophils [56].

However, with the exception of the up-regulation of LTB (lymphotoxin beta, also known as tumor necrosis factor C), classical pro-inflammatory molecules (such as cytokines of the tumor necrosis factor superfamily and the interleukins IL1, IL6, IL8) were not detected to be significantly deregulated. One possible explanation for this could be the investigated time-point, that is, Day 15 p.i., where we might have missed the acute inflammatory stage that is usually expected to show an immediate increase or decrease of the above listed molecules. In that case, the list of DEGs identified in the current study might be representing cellular factors important during the persistent stage of *M*. *agalactiae* infection. However, the lack of regulation of pro-inflammatory cytokines has also been previously reported in the blood of cattle during the acute stage of contagious bovine pleuropneumonia (CBPP) caused by *M*. *mycoides* subsp. *mycoides* [57]. This is of interest as inflammation is the most characteristic indication of CBPP. The lack of regulation for these genes in the present study could also be due to the dilution of cytokine-producing immune cells in the mammary tissue. In addition, the migration of immune cells from the site of infection to local inflamed lymph nodes might also contribute to the observed results [57].

In comparison to the udder transcriptome, the number and fold changes observed for the DEGs of the spleen transcriptome were comparatively lower. This is not unexpected as this experimental intramammary infection did not lead to systemic spread of *M*. *agalactiae*, not even to the left udder halves in two of the three sheep. Even in the one sheep where the infection spread to the left udder halves, the pathogen was isolated only after Day 7 p.i. and that too in relatively lower doses. Hence, the infection being self-contained in the inoculated right udder halves, showed a robust and significant immune response to control the spread of the pathogen as observed in the significantly up-regulated DEGs. Nevertheless, 22 significant DEGs were identified in spleen tissue, out of which 7 were down-regulated and the other 12 were up-regulated. NPTX1 encoding neuronal pentraxin is known to be a classical acute phase protein that is associated with the humoral arm of the innate immune response [58]. A similar upregulation of PTX3 gene encoding Pentraxin-3 protein was observed in bovine mammary tissue when challenged with *Staphylococcus aureus* and the recombinant protein was shown to bind to C1q, a subunit of the first component of the complement cascade [48]. Similarly, TFRC encoding transferrin receptor could be meaningful as transferrin is an iron-binding protein made in the liver, and has also been detected in similar proteomic analysis of mastitic milk [50]. Overall, the spleen transcriptome data could provide a useful basis for understanding the systemic responses to intra-mammary mycoplasma challenge. This is important not only because the susceptibility of an animal to develop mastitis likely depends on factors beyond the mammary tissues, but is also a vital factor affecting the persistence and systemic spread of the pathogen to new host niches [47].

When comparing DEGs from spleen with those of the udder, only the up-regulation (4-fold in spleen vs 63-fold in udder) of the class I histocompatibility antigen OLA-I could be detected in both tissue types. These molecules are found on the cell surface of all nucleated cells and are involved in the presentation of pathogen antigens to the immune system, in particular cytotoxic T cells, thus triggering an immediate immune response. The observed fold changes again indicate a stronger immune response at the infection site compared to the spleen. Detailed analyses of specific cell types will be necessary to further investigate the interplay between peripheral cells with the systemic immune system. Future studies should investigate the expression profiles of specific immune cells from local infection sites and systemic organs to gain insights into the mechanisms of antigen processing and mediation of adaptive immunity during the pathogenesis of *M*. *agalactiae*.

## Conclusion

In summary, this first comprehensive report of the local and systemic effects of the intramammary infection of *M*. *agalactiae* in sheep udders and spleen, respectively, should pave the way for further research to understand and define the exact host-pathogen interactions at the molecular level. However, we still need to characterize the mycoplasma components that induce such a robust host response in the inoculated udders. The study will be instrumental in developing new targets for disease intervention, and based on the obtained cues, could also help in the control of mastitis in general.

## Supporting Information

S1 Table RNA integrity numbers and RT-qPCR data. (XLS)

S2 Table Complete list of significantly enriched biological processes in *M*. *agalactiae*-infected mammary gland. The list of DEGs detected by RNA-Seq was investigated for significantly enriched biological processes. The significance level for each GO term is shown in the column ‘Term PValue’. The coloring of the individual biological processes reflects the degree of connectivity between the terms based on the similarity of their associated genes. Related GO terms are grouped into functional network groups (‘GO groups’) based on a statistical analysis of the associated genes. Each term can be included in multiple groups.
(XLS)
